# Announcement: SPRIG (A new Initiative in Bioinformatics Networking)

**DOI:** 10.1002/cfg.160

**Published:** 2002-04


### Announcement

### SPRIG (A New Initiative in Bioinformatics Networking)

SPRIG ('**S**pecialized **P**lant **R**esources for **I**nformatics and **G**enomics') is an initiative established last summer subsequent to the deliberations of plant-focused bioinformaticians attending the Intelligent Systems for Molecular Biology (ISMB) meeting in Copenhagen. SPRIG is a response to the observation that plant-focused bioinformaticians, unlike some of their counterparts in the human genome and model organism community, are few and far between, yet are subject to numerous yet common expectations from their client communities - databases, pipelines, user interfaces. The aim of SPRIG is to create **a virtual network of plant-focused bioinformaticians** to share information, protocols and resources, thus minimizing 'reinvention of the wheel' within the community.

The primary activity of SPRIG at present is the hosting of a web site, **http://bioinformatics.org/sprig**, exploiting the excellent public Bioinformatics.org site that provides facilities for bioinformatics groups wishing to host email list servers, discussion groups, web pages, FTP repositories and other useful project coordination resources. The group and web site is not species-specific but serves the whole plant community.

At present, the SPRIG site hosts a home page of basic information, a 'members' page of voluntarily contributed information about bioinformatics development activities of some group members, a general email list server, sprig-info@bioinformatics.org, that can serve as a broadcast point for informative announcements within the community. The site is also the current web home for the newly formed **'Plant Ontology Consortium'** (**http://bioinformatics/sprig/poc.html**) and associated email list, **sprig-CVO@bioinformatics.org**.

SPRIG is a 100% voluntary associated (and member resourced) consortium. Site organizers are looking towards the plant-focused bioinformatics community to specifically define what kinds of resources they would like to share and what kinds of activities they would like to sponsor. For example, there have been some discussions about coordinating SPRIG with a proposed 'SIGPLANT' special interest group within the International Society for Computational Biology.

Individuals or groups interested in contributing to SPRIG resources and efforts can directly sign up with the SPRIG project at **http://bioinformatics.org/** and/or contact Richard Bruskiewich (**r.bruskiewich@cgiar.org**), the current (volunteer) SPRIG coordinator.

